# Professional Etiquette

**Published:** 1855-09

**Authors:** 


*Professional Etiquette.*—If the duties which physicians owe to their own patients are the most imperative, their mutual relations and intercourse, professionally, are of the greatest importance, and never to be made subservient to mere selfish purposes.

We believe it is undeniable that our own medical community is remarkably free from those occurrences which constitute breaches of professional etiquette. This should be so everywhere, among those whose aim is to diffuse comfort and the means of health. A truly high standard of feeling and practice can hardly be maintained unless the honorable estimation of brethren and the real wish "to do unto others as we would they should do unto us," be cherished and regarded as the rule of action.

There are many ways of injuring a *confrère* besides overt detraction or explicit assertion of incompetency, &c. The shrug of the shoulders, or the incredulous aspect, if anything be said in his favor; the hints of small experience, want of opportunities for observation, personal peculiarities made prominent, &c., &c., often go further toward injuring another's prospects, than any specific charge detrimental in itself. If an older physician be asked about the qualifications of a younger one, it is clearly his duty to give him the benefit of his own good opinion if he honestly have one. A generous man would often go farther;—he would not allow mere *youth* to prevent success—but would say (what is now the truth,)that our younger physicians are nearly all highly educated—that their acquirements, in general, entitle them to the full confidence of the public, &c. It cannot diminish either the reputation or the income of the established physician to extend to the comparative neophyte a welcome, and a helping hand; it will only render the latter more deserving, if he be at all so, and will, through the years of weary waiting, inspire him with hope and give him an agreeable and elevated idea of his superiors in acquirements and experience.

The reverence for years and distinguished abilities is, we think, quite observable among our younger medical men, and while many may be mentioned who worthily inspire this sentiment, there is one for whom all seem, instinctively, to feel a genuine affection, and who affords us an eminent example of that professional courtesy which accords both to the advanced and the youthful practitioner, the consideration merited by each.

> Skill, judgment, science, and a spotless name
> Are not the only trophies of his fame :—
> While Honor's seal on every action shines,
> And Dignity with Gentleness combines ;—
> With learning's light the social virtues blend,
> And wondrous magic to his teachings lend—
> Peculiar beauty round his paths is shed,
> And young and old call blessings on his head !

There are those, we regret to say, who by thoughtlessness often—sometimes, it is to be feared, intentionally, throw out opinions or insinuations which materially injure the prospects of others who would fain rise in the scale of success as well as of merit. The rashness of the tongue is rarely ever more mischievous than in such instances.

It is hardly worth while to specify much; most physicians are well aware how much good or evil a word, "fitly" or otherwise spoken, is capable of doing. We should not judge one another too freely, nor should we draw conclusions from mere appearances. If a member of the profession be fond of its literature, or has been led to its cultivation by particular or fortuitous circumstances, hardly to be avoided, it does not follow that he has not a wish to practice, and others should not form such an opinion, much less promulgate it, unless they know it to be the fact *from his own mouth.* Otherwise, his laudable hope for a share in active professional labors and remuneration may be essentially thwarted: every man's word has influence; let it be exercised as he believes the persons under remark would themselves wish, at least with regard to their professional intentions.

It is by no means always true, that because a physician is living in comparatively easy circumstances, he is therefore careless of occupation. He may often, on the contrary, both earnestly desire, and really need it, to enable him to live. Because he has certain means, it should not be decided for him that he requires no more. Moreover, no one, especially no physician, should be an idle man—and can one, in these days, work for nothing? Too many do so for a long time. Professional etiquette demands that no one pronounce his fellow practitioner a drone until he is *very sure* of it. We conclude that every man in regular standing, who writes himself Doctor, and places an intimation to that effect upon his door or the corner of his house, thereby signifies his intention to offer his services to the public, and his wish not to be considered a "retired physician," as we once heard remarked of a practitioner in this city, very erroneously supposed to be rich and indifferent to practice, because he had been twice to Europe (upon both occasions devoting most of his time to medical and surgical studies) and did not live at the corner of a noisy thoroughfare!

We do not think it worth while to do more than refer to the paltry tricks and unscrupulous measures, most frequently intangible and secret, by which patients are sometimes filched from those who have long devoted themselves to their welfare. In many nameless ways is this done by some, but, as we conclude that such persons cannot understand even the meaning of the word etiquette, and are sure that they ignore, practically, the terms right and wrong, honor and injustice, we leave them to their shuffling and grovelling courses.

In consultations there is peculiar opportunity for the exercise of true courtesy. These meetings of physicians are of two sorts—necessary and unnecessary—pleasant or disagreeable. They are necessary whenever the practitioner in attendance is in doubt and anxiety about his patient, and if the conduct of all the parties concerned be what it should be, they are then pleasant and advantageous. Frequently, however, there is no real occasion for them: the family physician feels competent to manage the case, and apprehends no danger. If a consultation be forced upon him, it is somewhat trying; the adviser may make it less so—very probably even delightful; or he may, by his manner, increase any embarrassment or unpleasant feeling already awakened. He has it in his power to advance or injure the reputation and skill of the one whom he meets. In both instances, how much depends on the etiquette observed. Perfect ease, however, is wholly consistent with its due observance; and such consultations not infrequently result in the formation of life-long friendships, while those of an opposite character beget everlasting dislikes. The junior practitioner has his part to play in rendering consultations agreeable, and this duty should sit gracefully upon him. More importance attaches to these arrangements than may generally be believed. The rule is easy of application; let all, in their frequent and needful intercourse, refer each particular set of circumstances to themselves as the persons interested, and consult the *heart* as well as the reason in these matters; we will answer for the good effect which will be visible in the exercise of high professional etiquette.—*Boston Med. and Surg. Jour.*

